# Special Issue “Probiotics and Their Metabolism”: Editorial

**DOI:** 10.3390/microorganisms11030687

**Published:** 2023-03-08

**Authors:** Mohammed Kamal Salman, Gianluigi Mauriello

**Affiliations:** Department of Agricultural Sciences, University of Naples Federico II, 80055 Portici, Italy

As a general theory, the benefits of probiotics to human health and the prevention of disease are promoted by metabolites, which include antimicrobial compounds, short-chain fatty acids (SCFAs), organic acids, and bio-actives. Probiotics are subject to investigation in clinical settings as a therapy for many diseases, in particular inflammatory diseases. Research on the health-promoting effects of probiotics is still ongoing with many recent advances made in uncovering probiotics’ roles in the modulation of the gut microbiota; anti-oxidation, anti-inflammation, and curing of protozoal diseases have been reported [1,2]. Yet, the mechanism behind this modulation has still not been fully determined. Postbiotics, an area of research linked to probiotics, was recently redefined by the International Scientific Association of Probiotics and Prebiotics (ISAPP) as “preparation of inanimate microorganisms and/or their components that confers a health benefit on the host”. A wide range of postbiotic compounds such as proteins, exopolysaccharides, SCFAs, lactic acid, dopamine, serotonin, histamines, and vitamins have been postulated to induce positive effects on gut microbiota, intestinal barrier function, and inflammatory response [3]. Due to the complex nature of host–microbiome interaction and the process of bacterial metabolism, there is still a need to investigate the molecular and biological mechanism of metabolic by-products of probiotics. Furthermore, the term ‘probiotic’ is extending to include more probiotic species next to the well-known lactic acid bacteria and bifidobacteria; such species are commonly referred to as next/new-generation probiotics (NGPs). NGPs have a promising effect in the amelioration of inflammatory diseases, metabolic syndrome, and obesity [4,5].

This Special Issue, “Probiotics and Their Metabolism”, presents the findings of three research papers and one review on the health benefits of some probiotics and their metabolites. Bacteria develop different metabolic pathways when they grow in mono-culture or co-culture environments. The studies currently available in the literature do not fully elaborate on how the metabolic pathways differ when bacteria grow in mono- and multi-culture environments. This is due to the challenge of modeling the growth medium, however. In a study by Ulmer et al. [6], a synthetic growth medium was developed to monitor the growth of dairy starter culture (*Streptococcus thermophilus* and *Lactobacillus bulgaricus* in mono- and co-cultures) and amino acid production. The results showed that amino acids release was not the same in mono- and co-cultures. The histidine levels were higher in the co-culture and there was a depletion in isoleucine and arginine.

Polyamines intake is associated with health-promoting effects and dietary intake of polyamine is not related to carcinogenesis [7]. *Latilactobacillus curvatus* KP 3–4 isolated from fermented Japanese product (Kabura-zushi) was found to be able to produce abundant levels of putrescine extracellularly, which suggests the probiotication of a novel food product with *Latilactobacillus curvatus* KP 3–4 isolate as a polyamine-producing strain [8]. P75, a *Lacticaseibacillus rhamnosus* GG-derived protein, is a bioactive probiotic metabolite. In a study by Kang et al. [9], P75 carried on *Bacillus* spores stimulated a wide genomic response in HT-29 cells which are involved in biological processes such as epithelial cell development. In the review study, it was shown that *Limosilactobacillus reuteri* displayed a strain-dependent ameliorative effect on metabolic syndrome, gut dysbiosis, the gut–brain axis, and hepatic disorders. This is characterized by the metabolites of *Limosilactobacillus reuteri,* which include reuterin, exopolysaccharides, surface proteins, reutericyclin, and organic acids [10]. The future studies should be focused on conducting clinical trials to investigate the biological effect of combining different probiotic strains and their metabolites. In addition, there is still a gap in understanding the probiotic and NGP candidates–pathogenic bacteria interaction and how probiotics/postbiotics can disrupt the expression of virulence factors in the human gut, which are controlled by the mechanism of quorum sensing (QS) and the bacterial cell-to-cell communication that is induced by chemical molecules (auto-inducers).
